# 5-Bromo-1*H*-thieno[2,3-*d*]imidazole

**DOI:** 10.1107/S1600536810027224

**Published:** 2010-07-14

**Authors:** Fen Wang, Sacha Ninkovic, Michael Collins, Curtis Moore, Arnold L. Rheingold, Alex Yanovsky

**Affiliations:** a Pfizer Global Research and Development, La Jolla Labs, 10770 Science Center Drive, San Diego, CA 92121, USA; b Department of Chemistry and Biochemistry, University of California, San Diego, 9500 Gilman Drive, La Jolla, CA 92093, USA

## Abstract

The crystal structure of the title compound, C_5_H_3_BrN_2_S, shows that bromination of 1*H*-thieno[2,3-*d*]imidazole with *N*-bromo­succinimide in acetonitrile occurs at position 5 of the bicyclic system. The mol­ecule is almost planar, with a mean deviation of 0.015 Å from the least-squares plane through all the non-H atoms. In the crystal, N—H⋯N hydrogen bonds link the mol­ecules into infinite *C*(4) chains running along [101].

## Related literature

For a related structure involving the thieno[2,3-*d*]imidazole fragment, see: Busetti *et al.* (1989 ▶).
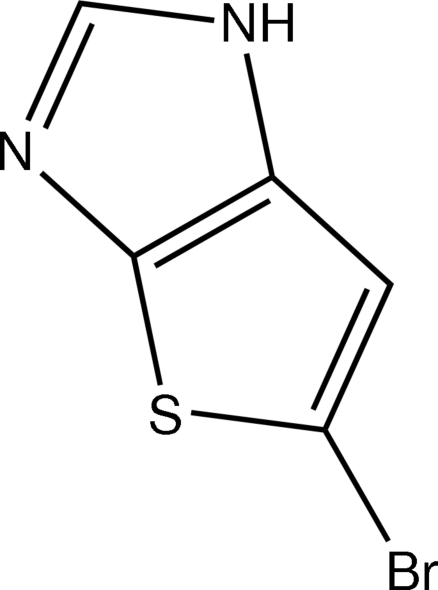


## Experimental

### 

#### Crystal data

C_5_H_3_BrN_2_S
*M* *_r_* = 203.06
Monoclinic, 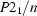

*a* = 3.8917 (11) Å
*b* = 17.118 (5) Å
*c* = 9.405 (3) Å
β = 91.359 (3)°
*V* = 626.4 (3) Å^3^
*Z* = 4
Mo *K*α radiation
μ = 6.79 mm^−1^
*T* = 100 K
0.32 × 0.18 × 0.09 mm

#### Data collection

Bruker APEXII CCD diffractometer
Absorption correction: multi-scan (*SADABS*; Bruker, 2001 ▶) *T*_min_ = 0.220, *T*_max_ = 0.580
9691 measured reflections
1441 independent reflections
1270 reflections with *I* > 2σ(*I*)
*R*_int_ = 0.043

#### Refinement

*R*[*F* ^2^ > 2σ(*F* ^2^)] = 0.036
*wR*(*F* ^2^) = 0.098
*S* = 1.08
1441 reflections
83 parameters
H-atom parameters constrained
Δρ_max_ = 1.41 e Å^−3^
Δρ_min_ = −0.81 e Å^−3^

### 

Data collection: *APEX2* (Bruker, 2007 ▶); cell refinement: *SAINT* (Bruker, 2007 ▶); data reduction: *SAINT*; program(s) used to solve structure: *SHELXS97* (Sheldrick, 2008 ▶); program(s) used to refine structure: *SHELXL97* (Sheldrick, 2008 ▶); molecular graphics: *SHELXTL* (Sheldrick, 2008 ▶); software used to prepare material for publication: *SHELXTL*.

## Supplementary Material

Crystal structure: contains datablocks global, I. DOI: 10.1107/S1600536810027224/hb5547sup1.cif

Structure factors: contains datablocks I. DOI: 10.1107/S1600536810027224/hb5547Isup2.hkl

Additional supplementary materials:  crystallographic information; 3D view; checkCIF report

## Figures and Tables

**Table 1:** Hydrogen-bond geometry (Å, °)

*D*—H⋯*A*	*D*—H	H⋯*A*	*D*⋯*A*	*D*—H⋯*A*
N1—H1*N*⋯N2^i^	0.88	2.04	2.903 (4)	165

Symmetry code: (i) 
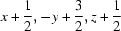
.

**Fig. 1.**
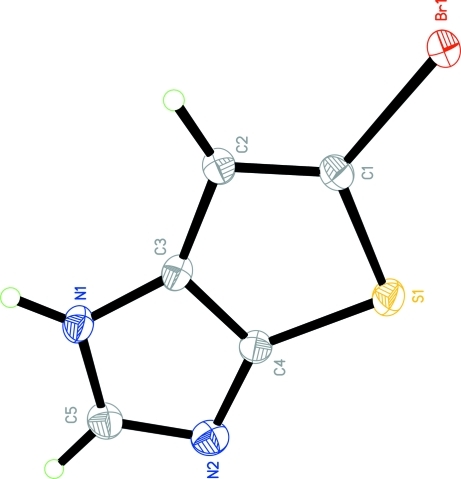
Molecular structure of (I), showing 50% probability displacement ellipsoids. H atoms are drawn as circles with arbitrary small radius.

**Fig. 2.**
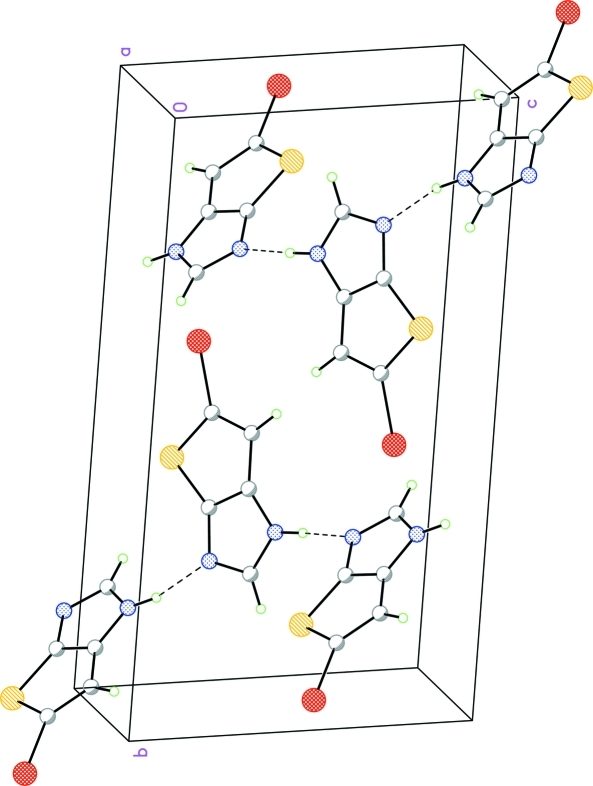
Packing diagram of (I) viewed approximately down the a axis.

## supplementary crystallographic information

### Comment

Bromination of 1*H*-thieno[2,3-*d*]imidazole by bromosuccinimide in acetonitrile may occur either at position 2,5 or 6. The present X-ray study showed that substitution occurs in fact at position 5; the molecular structure of the title compound, representing the major product of bromination is shown in Fig. 1.

The molecule of the title compound is planar; maximum deviation of the Br1 atom from the mean plane of all non-H atoms of the molecule is equal to 0.029 (3) Å. To the best of our knowledge, this is the first structural study of thieno[2,3-*d*]imidazole derivative with the isolated bicyclic system. In the only closely related molecule, studied by the single-crystal X-ray diffraction earlier (Busetti *et al.*, 1989), the thieno[2,3-*d*]imidazole system is fused with the benzene ring, thus forming a tricyclic molecule. The geometry of the thienoimidazole fragment of the tricyclic molecule is very similar to that of the title compound.

There is one symmetry independent intermolecular H-bond in the structure (Table 1), which is responsible for the formation of infinite chains of molecules running along the [101] direction of the crystal (Fig. 2).

### Experimental

To a solution of 1*H*-thieno[2,3-*d*]imidazole (16 mg, 0.13 mmol) in 0.75 ml of acetonitrile at 0°C was added dropwise *N*-bromosuccinimide (21 mg, 0.12 mmol) in 0.25 ml of acetonitrile. The reaction mixture was stirred at 0°C for 0.5 hr. The reaction mixture was diluted with 10 ml of 1 N NaOH, and the aqueous layer was extracted with EtOAc (3 × 15 ml). The organic layers were combined, dried over Na_2_SO_4_, filtered and concentrated. The product was purified by flash chromatography (silica gel, 0–70% EtOAc/heptane) to give 22 mg (81%) 5-bromo-1*H*-thieno[2,3-*d*]imidazole as an off white solid.

Light brown needles of (I) were grown by slow evaporation of a 20/20/60 methanol/dichloromethane/toluene solution

### Refinement

All H atoms were placed in geometrically calculated positions (N—H 0.88 Å, C—H 0.95 Å) and included in the refinement in the riding motion approximation. The *U*_iso_(H) were set to 1.2*U*_eq_ of the carrying atom. The highest residual peak of 1.41 e/Å^3^ is located at 0.91 Å from the Br1 atom.

### Crystal data

**Table:**

C_5_H_3_BrN_2_S	*F*(000) = 392
*M**_r_* = 203.06	*D*_x_ = 2.153 Mg m^−^^3^
Monoclinic, *P*2_1_/*n*	Mo *K*α radiation, λ = 0.71073 Å
Hall symbol: -P 2yn	Cell parameters from 4198 reflections
*a* = 3.8917 (11) Å	θ = 2.4–27.4°
*b* = 17.118 (5) Å	µ = 6.79 mm^−^^1^
*c* = 9.405 (3) Å	*T* = 100 K
β = 91.359 (3)°	Needle, light brown
*V* = 626.4 (3) Å^3^	0.32 × 0.18 × 0.09 mm
*Z* = 4	

### Data collection

**Table:**

Bruker APEX CCD diffractometer	1441 independent reflections
Radiation source: fine-focus sealed tube	1270 reflections with *I* > 2σ(*I*)
graphite	*R*_int_ = 0.043
φ and ω scans	θ_max_ = 28.2°, θ_min_ = 2.4°
Absorption correction: multi-scan (*SADABS*; Bruker, 2001)	*h* = −5→5
*T*_min_ = 0.220, *T*_max_ = 0.580	*k* = −22→21
9691 measured reflections	*l* = −11→12

### Refinement

**Table:**

Refinement on *F*^2^	Primary atom site location: structure-invariant direct methods
Least-squares matrix: full	Secondary atom site location: difference Fourier map
*R*[*F*^2^ > 2σ(*F*^2^)] = 0.036	Hydrogen site location: inferred from neighbouring sites
*wR*(*F*^2^) = 0.098	H-atom parameters constrained
*S* = 1.08	*w* = 1/[σ^2^(*F*_o_^2^) + (0.0585*P*)^2^ + 0.9841*P*] where *P* = (*F*_o_^2^ + 2*F*_c_^2^)/3
1441 reflections	(Δ/σ)_max_ < 0.001
83 parameters	Δρ_max_ = 1.41 e Å^−^^3^
0 restraints	Δρ_min_ = −0.81 e Å^−^^3^

### Special details

**Table:**

Geometry. All e.s.d.'s (except the e.s.d. in the dihedral angle between two l.s. planes) are estimated using the full covariance matrix. The cell e.s.d.'s are taken into account individually in the estimation of e.s.d.'s in distances, angles and torsion angles; correlations between e.s.d.'s in cell parameters are only used when they are defined by crystal symmetry. An approximate (isotropic) treatment of cell e.s.d.'s is used for estimating e.s.d.'s involving l.s. planes.
Refinement. Refinement of *F*^2^ against ALL reflections. The weighted *R*-factor *wR* and goodness of fit *S* are based on *F*^2^, conventional *R*-factors *R* are based on *F*, with *F* set to zero for negative *F*^2^. The threshold expression of *F*^2^ > σ(*F*^2^) is used only for calculating *R*-factors(gt) *etc*. and is not relevant to the choice of reflections for refinement. *R*-factors based on *F*^2^ are statistically about twice as large as those based on *F*, and *R*- factors based on ALL data will be even larger.

### Fractional atomic coordinates and isotropic or equivalent isotropic displacement parameters (Å^2^)

**Table:**

	*x*	*y*	*z*	*U*_iso_*/*U*_eq_	
Br1	0.78267 (9)	0.43247 (2)	0.25169 (4)	0.01969 (18)	
S1	0.4398 (2)	0.58614 (6)	0.13967 (10)	0.0204 (3)	
N1	0.4603 (8)	0.71893 (18)	0.4614 (3)	0.0186 (7)	
H1N	0.5141	0.7276	0.5515	0.022*	
N2	0.2480 (8)	0.7397 (2)	0.2389 (3)	0.0212 (7)	
C1	0.6366 (9)	0.5359 (2)	0.2806 (4)	0.0178 (7)	
C2	0.6719 (9)	0.5760 (2)	0.4074 (4)	0.0159 (7)	
H2	0.7747	0.5568	0.4932	0.019*	
C3	0.5239 (9)	0.6521 (2)	0.3853 (4)	0.0169 (7)	
C4	0.3908 (9)	0.6662 (2)	0.2503 (4)	0.0168 (7)	
C5	0.2976 (10)	0.7690 (2)	0.3697 (4)	0.0206 (8)	
H5	0.2262	0.8200	0.3960	0.025*	

### Atomic displacement parameters (Å^2^)

**Table:**

	*U*^11^	*U*^22^	*U*^33^	*U*^12^	*U*^13^	*U*^23^
Br1	0.0235 (2)	0.0164 (3)	0.0190 (3)	0.00184 (12)	−0.00180 (14)	−0.00335 (13)
S1	0.0251 (5)	0.0207 (5)	0.0153 (5)	0.0007 (3)	−0.0021 (4)	−0.0008 (4)
N1	0.0240 (16)	0.0179 (16)	0.0140 (16)	−0.0007 (12)	−0.0001 (12)	−0.0003 (13)
N2	0.0225 (16)	0.0183 (18)	0.0227 (18)	−0.0007 (11)	−0.0005 (13)	0.0031 (13)
C1	0.0189 (17)	0.0149 (18)	0.0196 (19)	−0.0004 (13)	0.0011 (13)	−0.0008 (15)
C2	0.0201 (17)	0.0141 (17)	0.0137 (18)	−0.0012 (13)	0.0033 (13)	0.0018 (13)
C3	0.0195 (17)	0.0171 (18)	0.0140 (18)	−0.0024 (13)	−0.0009 (13)	0.0002 (14)
C4	0.0204 (17)	0.0140 (17)	0.0161 (19)	−0.0002 (14)	−0.0006 (13)	0.0018 (14)
C5	0.0258 (19)	0.0154 (18)	0.021 (2)	0.0007 (14)	0.0012 (15)	0.0020 (15)

### Geometric parameters (Å, °)

**Table:**

Br1—C1	1.882 (4)	N2—C4	1.378 (5)
S1—C4	1.734 (4)	C1—C2	1.379 (5)
S1—C1	1.742 (4)	C2—C3	1.438 (5)
N1—C5	1.362 (5)	C2—H2	0.9500
N1—C3	1.375 (5)	C3—C4	1.381 (5)
N1—H1N	0.8800	C5—H5	0.9500
N2—C5	1.338 (5)		
			
C4—S1—C1	89.20 (18)	C3—C2—H2	126.4
C5—N1—C3	106.3 (3)	N1—C3—C4	105.3 (3)
C5—N1—H1N	126.8	N1—C3—C2	139.1 (3)
C3—N1—H1N	126.8	C4—C3—C2	115.6 (3)
C5—N2—C4	102.8 (3)	N2—C4—C3	111.9 (3)
C2—C1—S1	116.5 (3)	N2—C4—S1	136.4 (3)
C2—C1—Br1	124.6 (3)	C3—C4—S1	111.6 (3)
S1—C1—Br1	118.9 (2)	N2—C5—N1	113.6 (3)
C1—C2—C3	107.1 (3)	N2—C5—H5	123.2
C1—C2—H2	126.4	N1—C5—H5	123.2

### Hydrogen-bond geometry (Å, °)

**Table:**

*D*—H···*A*	*D*—H	H···*A*	*D*···*A*	*D*—H···*A*
N1—H1N···N2^i^	0.88	2.04	2.903 (4)	165

Symmetry codes: (i) *x*+1/2, −*y*+3/2, *z*+1/2.
